# Impact of Postoperative Weight-Bearing Protocols on Prognosis in Geriatric Hip Fracture Patients: A Systematic Review and Meta-Analysis

**DOI:** 10.3390/jcm15103912

**Published:** 2026-05-19

**Authors:** Shanbin Xu, Feng Gao, Yimin Chen, Gang Liu, Kangzu Peng, Jing Zhang, Liunan Chen, Yew Lok Woo, Ronald Man Yeung Wong, Maoyi Tian, Xinbao Wu, Minghui Yang

**Affiliations:** 1 Department of Orthopedics and Traumatology, Beijing Jishuitan Hospital, Capital Medical University, 31 Xinjiekou East Road, Xicheng District, Beijing 100035, China; 2 Fourth School of Clinical Medicine, Peking University, Beijing 100035, China; 3 National Center for Orthopaedics, Beijing 100035, China; 4 School of Public Health, Harbin Medical University, Harbin 150081, China; 5 Department of Orthopaedic Surgery, Singapore General Hospital, Singapore 169608, Singapore; 6 Department of Orthopaedics and Traumatology, The Chinese University of Hong Kong, Hong Kong, China

**Keywords:** hip fracture, elderly, weight-bearing, mortality

## Abstract

**Background**: Clinical controversy persists regarding the optimal weight-bearing strategy for elderly patients following hip fractures. Whilst early unrestricted weight-bearing may improve functional outcome and reduce the risk of bed-related complications, concerns about implant stability and failure often lead clinicians to adopt restricted weight-bearing protocols. To address this, we conducted a systematic review and meta-analysis to identify the effects of unrestricted weight-bearing compared with restricted weight-bearing on clinical outcomes in this patient population. We hypothesized that unrestricted weight-bearing may be associated with lower all-cause mortality without increasing postoperative complications, reoperation rates or length of hospital stay (LOS). **Methods**: This systematic review was conducted based on a study protocol registered on the PROSPERO platform and reported strictly in accordance with the PRISMA guidelines. We included clinical studies involving patients aged ≥65 years with hip fractures undergoing surgical treatment that directly compared the effects of different postoperative weight-bearing strategies on outcomes. Patients were further classified into unrestricted and restricted weight-bearing groups according to the postoperative weight-bearing protocols reported in each study. The primary outcome was all-cause mortality. Secondary outcomes included postoperative complications, reoperation rates, and LOS. A random-effects model was used for meta-analysis. Dichotomous variables were expressed as risk ratios (RRs), continuous variables as mean differences (MDs), and study heterogeneity was assessed using the I^2^ statistic. The certainty of evidence of each outcome was assessed by using the Grading of Recommendations Assessment, Development and Evaluation (GRADE) approach. **Results**: Ten studies (one randomized controlled trial and nine cohort studies) were included with 5806 patients in total. Extra-capsular fractures (intertrochanteric/subtrochanteric fractures) were the most common, with 3694 patients, followed by femoral neck fractures, with 1929 patients. Unrestricted weight-bearing was significantly associated with lower long-term mortality compared with restricted weight-bearing (RR = 0.67, 95% CI 0.51–0.88, *p* = 0.004, I^2^ = 34%; 95% PI 0.52–0.83), with an absolute risk difference of −0.10%. Short-term mortality did not differ significantly in the primary analysis (RR = 0.58, 95% CI 0.14–2.34, *p* = 0.44, I^2^ = 70%; 95% PI 0.00–126.98). Furthermore, the corresponding absolute risk difference was only −0.03%. No significant differences were observed for short-term complications, long-term complications, reoperation risk, or LOS between the two groups (all *p* > 0.05). GRADE assessment showed low certainty of evidence for long-term mortality and short-term complications, and very low certainty of evidence for the remaining outcomes. **Conclusions**: This meta-analysis suggests that unrestricted weight-bearing may be a feasible postoperative rehabilitation approach in selected patients. However, the results should be interpreted with caution. Further well-designed prospective studies are required to confirm these findings.

## 1. Introduction

In the context of global population aging and increasing life expectancy, hip fractures in the elderly have become a serious challenge to public health [1,2]. It was reported that the global standardized incidence rate of hip fractures among older adults was estimated to be 948.81 per 100,000 population in 2021 [3]. Consistent with the global trend, high incidence rates have also been reported across different regions. In the United States, approximately 300,000 older adults sustain a hip fracture each year [4], while in Europe, an estimated 827,000 fragility hip fractures occurred in 2019 [5]. In Asia, the standardized incidence rate of hip fractures has been reported to range from 90 to 318 per 100,000 population [6]. Importantly, this burden is not limited to high fracture incidence. These fractures also have high mortality and disability rates, and are often referred to as ‘the last fracture of life’ [7,8]. Therefore, geriatric hip fractures severely affect the quality of life for older adults and impose a substantial economic and social burden on healthcare systems worldwide [9,10,11].

It is widely recognized that timely surgical intervention is crucial for improving the prognosis of elderly patients with hip fractures [12]. However, the patient’s final outcomes are not determined by surgery alone. A growing body of clinical evidence indicates that, on the basis of successful surgical procedures, an evidence-based postoperative weight-bearing strategy serves as an essential complement to the treatment of elderly hip fractures [13]. An appropriate weight-bearing protocol, combined with successful surgery, jointly promotes functional recovery, restoration of mobility, and improved survival outcomes for patients [14,15].

However, there remains considerable controversy regarding the optimal weight-bearing regimen following hip fracture surgery in the elderly. Proponents of unrestricted weight-bearing argue that it can promote early mobilization, reduce prolonged bed rest, and may prevent life-threatening complications such as pneumonia, pressure ulcers, and deep vein thrombosis (DVT) [13,14,16,17]. Furthermore, available evidence suggests that weight-bearing as tolerated does not increase implant failure or worsen functional outcomes compared with restricted protocols [15]. However, proponents of restricted weight-bearing emphasize the potential mechanical risks of rapid unrestricted weight-bearing, especially in patients with unstable fracture patterns, poor bone quality, or uncertain fixation stability [14,18]. They argue that partial or non-weight-bearing may reduce the risk of fixation failure, thereby safeguarding long-term outcomes [16,18,19,20,21]. These conflicting views highlight the long-standing uncertainty surrounding postoperative weight-bearing protocols in clinical practice.

To address this gap, we conducted a systematic review and meta-analysis to identify the effects of unrestricted weight-bearing compared with restricted weight-bearing on all-cause mortality, postoperative complications, reoperation rates, and length of hospital stay (LOS) amongst patients aged 65 years and older undergoing surgery for hip fractures. We hypothesized that unrestricted weight-bearing may be associated with lower all-cause mortality without increasing postoperative complications, reoperation rates or LOS. We hope that this study will provide evidence-based medicine for clinical decision-making, thereby optimizing postoperative management and improving patient outcomes.

## 2. Methods

This systematic review was conducted according to a pre-specified protocol registered with the International Prospective Register of Systematic Reviews (PROSPERO number: CRD420251081686). The reporting of this systematic review and meta-analysis adheres to the Preferred Reporting Items for Systematic Reviews and Meta-Analyses (PRISMA) statement and the completed PRISMA checklist is provided in the Supplementary Table S1.

### 2.1. Search Strategy

We searched the PubMed, Embase, Web of Science, and Cochrane Library databases from the inception of each database up to 8 July 2025. Keyword searches employed a combination of ‘hip fracture’ and ‘weight-bearing’. Specific search strategies were developed for each database, detailed in Supplementary Tables S2–S5 [22]. We also screened reference lists of identified studies and relevant review articles to supplement additional eligible studies.

### 2.2. Selection Criteria

We included clinical studies meeting all of the following criteria: (1) participants aged ≥65 years undergoing surgical treatment for hip fractures; (2) studies comparing different postoperative weight-bearing strategies; and (3) reporting at least one outcome measure, including mortality, postoperative complications, reoperation, or LOS. Studies involving pathological fractures, prior hip surgery, or patients with previous hip fractures were excluded. Non-original literature (such as reviews, letters, case reports, or technical notes) and non-English publications were also excluded. Previous evidence suggested that excluding non-English studies is unlikely to lead to biased results [23]. Duplicate records were removed.

The search results were imported into EndNote software (version 20, Clarivate Analytics, Philadelphia, PA, USA) for literature management, to facilitate the screening and selection of studies [24]. Two reviewers (SX, FG) independently assessed the suitability of studies. Titles and abstracts were initially screened, followed by full-text review of potentially relevant articles. Disagreements were resolved through discussion. Where necessary, a third reviewer (Y.C.) provided a final decision.

### 2.3. Data Extraction

Two reviewers (S.X. and F.G.) independently extracted data from all included studies. Extracted data were: corresponding author, year of publication, country of study, study design, sample size, mean age, proportion of females, fracture type, postoperative weight-bearing protocol, follow-up duration, and outcome measures of interest.

The primary outcome measure was all-cause mortality. Secondary outcome measures included postoperative complications, reoperation rates, and LOS. To enhance comparability between studies with different follow-up protocols, all-cause mortality and postoperative complications were categorized as short-term or long-term outcomes based on assessment time. Short-term outcomes were defined as those assessed during hospitalization or within 30 days postoperatively. Long-term outcomes were defined as those assessed ≥ 1 year postoperatively.

In this study, we have reclassified postoperative weight-bearing strategies. Weight-bearing protocols defined in the original articles as unrestricted weight-bearing, weight-bearing as tolerated, or full weight-bearing were all categorized as unrestricted weight-bearing in our analysis. Similarly, protocols defined as restricted weight-bearing, partial weight-bearing, touch weight-bearing, and non-weight-bearing in the original articles were all classified as restricted weight-bearing in our study. Disagreements were resolved by a third reviewer (YC).

### 2.4. Assessment of Quality and Bias

The methodological quality and risk of bias of included studies were independently assessed by two reviewers (S.X. and F.G.). Observational studies were evaluated using the Newcastle–Ottawa Scale (NOS), with scores of 7 or higher indicating high quality [25]. Randomized controlled trials were assessed using the Cochrane Risk of Bias Tool [26]. Disagreements were resolved by a third author (Y.C.) or with the involvement of senior authors (J.Z. and M.Y.).

### 2.5. Statistical Analysis

We conducted meta-analyses using Review Manager (RevMan, version 5.3; The Cochrane Collaboration, Copenhagen, Denmark) and R software (version 4.1.1; R Foundation for Statistical Computing, Vienna, Austria). Given the anticipated clinical and methodological heterogeneity amongst studies, we pre-specified the use of a random-effects model. For dichotomous outcomes (mortality, complications, and reoperation rates), pooled effect estimates were expressed as risk ratios (RRs) with 95% confidence intervals (CIs). For continuous outcome measures (LOS), results were summarized as mean differences (MDs) with 95% CI. Prediction intervals (PIs) were calculated for random-effects models to evaluate the heterogeneity and reflect the expected range of true effects across different settings. In addition, absolute risk differences were also reported. Pooled estimates were derived from unadjusted effect measures calculated from the reported event counts in the original studies, as adjusted estimates were rarely reported in each outcome and could not be meaningfully pooled. Statistical heterogeneity was assessed using the I^2^ statistic, with I^2^ > 50% indicating substantial heterogeneity. All analyses employed two-sided testing, with *p* < 0.05 considered statistically significant. When more than 10 studies are included, a funnel plot will be employed to assess potential publication bias. Where applicable, a leave-one-out and fixed-effect models sensitivity analysis will be conducted. Where applicable, subgroup analyses, meta-regression and influence diagnostics will be conducted to explore potential sources of heterogeneity.

### 2.6. Certainty of Evidence

Two independent authors (S.X. and F.G.) assessed the certainty of evidence for each pooled outcome using the Grading of Recommendations Assessment, Development and Evaluation (GRADE) approach [27]. The certainty of evidence was graded as very low, low, moderate, or high by evaluating study design, risk of bias, inconsistency, indirectness, imprecision, and publication bias. As most included studies were observational studies, the initial certainty of evidence was considered low. The three domains for rating up observational evidence, including a large magnitude of effect, dose–response gradient, and plausible residual confounding that would reduce the observed effect, were also considered. Disagreements were resolved through discussion or consultation with a third author (Y.C.).

## 3. Results

### 3.1. Search Results

**Figure 1:**
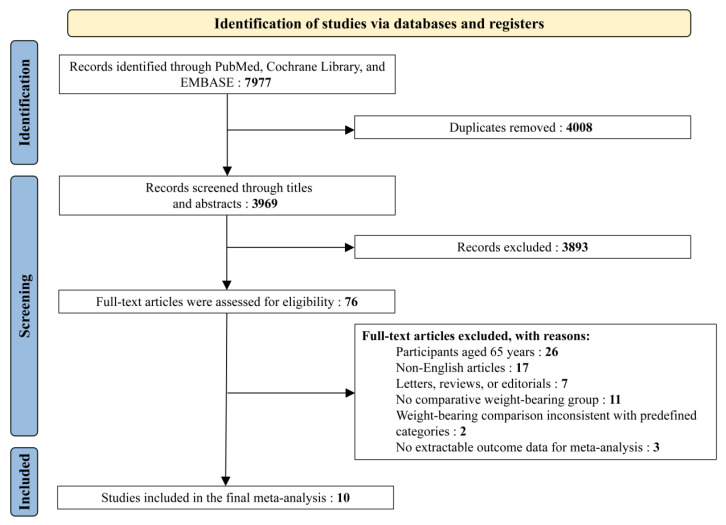
Flow of studies through the systematic review.

The initial search identified 7977 records, including 1575 from PubMed, 423 from the Cochrane Library, 3253 from Embase, and 2726 from Web of Science. After removing 4008 duplicate records, 3969 records proceeded to the screening stage. Following title and abstract screening, 3893 records were excluded, leaving 76 full-text articles for review. Following full-text assessment, 66 studies were excluded for the following reasons: participants aged under 65 years (*n* = 26), non-English publications (*n* = 17), letters/reviews/editorials (*n* = 7), absence of a comparison group (*n* = 11), weight-bearing classification not compatible with predefined definitions (*n* = 2), and inability to extract data suitable for meta-analysis (*n* = 3). Ultimately, 10 studies met the inclusion criteria and were incorporated into the final meta-analysis (Figure 1) [14,15,21,28,29,30,31,32,33,34].

### 3.2. Clinical Characteristics

Key characteristics of the included studies are summarized in Table 1. These 10 studies were published between 2014 and 2024, with research sites located in Turkey, Australia, China, Switzerland, Israel, and Spain. The included studies comprise 1 randomized controlled trial involving 85 participants and 9 cohort studies (retrospective or prospective) involving a total of 5721 participants. Across the studies, the average age of patients ranged from 76.7 to 85.5 years. The majority of research cohorts were predominantly female, with female patients accounting for between 49.4% and 84% of the total. The types of hip fractures varied across studies, encompassing femoral neck and/or intertrochanteric fractures, with some cohorts also including subtrochanteric fractures. Overall, fracture types were predominantly extra-capsular (intertrochanteric/subtrochanteric fractures), involving 3694 patients, followed by femoral neck fractures, with 1929 patients. In addition, internal fixation was the most common surgical strategy for treating hip fractures, applied to 4309 patients. Postoperative weight-bearing protocols also differed between studies. Three studies compared full weight-bearing with partial weight-bearing [28,29,30]. Three studies compared weight-bearing as tolerated with non-weight-bearing [14,15,32]. Two studies compared weight-bearing as tolerated with restricted/non-weight-bearing [21,33]. One study compared full weight-bearing with non-weight-bearing [34]. Another study employed a three-group design (full weight-bearing vs. partial weight-bearing vs. non-weight-bearing) [31].

### 3.3. Results of Quality and Risk-of-Bias Assessment

The NOS scores for the nine non-randomized studies ranged from 6 to 9. Notably, five studies scored >7. The remaining four studies scored 6–7. Overall, the included observational evidence demonstrated moderate to high study quality. The sole randomized controlled trial was assessed using the Cochrane risk of bias tool, with overall risk rated as high risk (Table 2).

Risk of bias was assessed using the Newcastle–Ottawa Scale. A higher total score indicated a lower risk of bias. A total score less than or equal to 5 indicates a high risk of bias.

The Cochrane risk of bias tool was used to assess the quality of a randomized controlled trial.

### 3.4. Mortality

**Figure 2:**
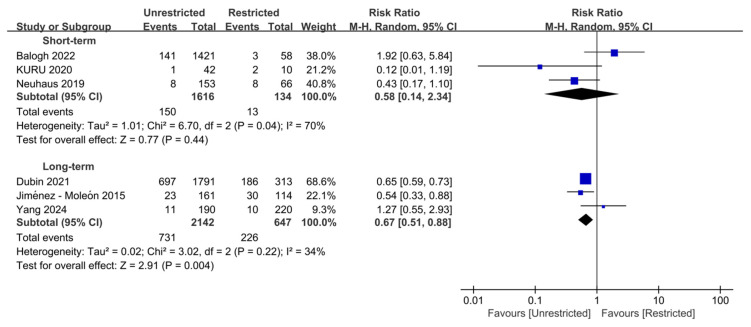
Stratified analysis of all-cause mortality according to follow-up duration [14,21,29,30,31,32]. Forest plot of unadjusted relative risks for all-cause mortality comparing unrestricted with restricted weight-bearing after hip fracture in the elderly, assessed in-hospital or at 30 days (short-term) and at ≥1 year (long-term). Unrestricted weight-bearing was associated with a lower risk of long-term mortality, whereas no significant difference was observed for short-term mortality. Abbreviations: CI, confidence interval; Tau^2^, between-study variance; Chi^2^, chi-square test statistic for heterogeneity; df, degrees of freedom; I^2^, inconsistency statistic.

All-cause mortality outcome data were derived from six studies and analyzed in stratified analyses according to pre-specified follow-up time windows (Figure 2). The meta-analysis of three studies compared short-term mortality between unrestricted and restricted weight-bearing protocols [21,29,30]. Random-effects model found no significant differences between the two groups in short-term mortality (RR = 0.58, 95% CI 0.14–2.34; *p* = 0.44), with high heterogeneity (I^2^ = 70%) and a wide PI crossing the null value (95% PI 0.00–126.98). Consistent with these findings, the corresponding absolute risk difference was only −0.03%. Further sensitivity analysis, excluding the Balogh study, revealed a significant reduction in heterogeneity (I^2^ = 3%) (Supplementary Figure S1). Pooled effects suggested unrestricted weight-bearing was associated with a significant decrease in short-term mortality (RR = 0.36, 95% CI 0.15–0.85; *p* = 0.02).

The meta-analysis of three studies compared long-term mortality between unrestricted and restricted weight-bearing protocols [14,31,32]. The pooled analysis demonstrated that unrestricted weight-bearing was associated with a 33% reduction in the risk of long-term all-cause mortality (95% CI 0.51–0.88, *p* = 0.004; 95% PI 0.52–0.83), with an acceptable level of heterogeneity (I^2^ = 34%). Furthermore, the corresponding absolute risk difference can reach −0.10%.

### 3.5. Complications

**Figure 3:**
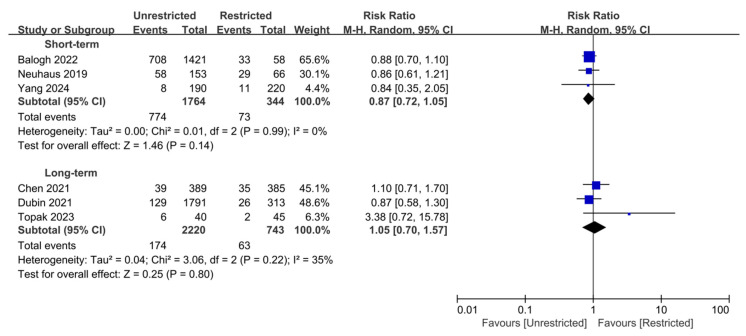
Stratified analysis of postoperative complications according to follow-up duration [14,15,21,28,30,31]. Forest plot of unadjusted relative risks for postoperative complications comparing unrestricted with restricted weight-bearing after hip fracture in the elderly, assessed in-hospital or at 30 days (short-term) and at ≥1 year (long-term). No significant difference in short-term or long-term complication risk was observed between the two weight-bearing strategies. Abbreviations: CI, confidence interval; Tau^2^, between-study variance; Chi^2^, chi-square test statistic for heterogeneity; df, degrees of freedom; I^2^, inconsistency statistic.

Figure 3 presents the analysis results for postoperative complications across six studies stratified by follow-up time windows. Short-term complications primarily include deep vein thrombosis, pneumonia, urinary tract infections, delirium, and falls during hospitalization. Some studies have also reported serious adverse events such as cerebrovascular accidents, myocardial infarction, heart failure, respiratory failure, and renal insufficiency. In contrast, long-term complications chiefly encompass varus collapse, screw cut-out, stress fractures of the femoral shaft, and non-union, with cerebrovascular accident and infection occasionally reported (Supplementary Table S6). In three studies, researchers used random-effects models to analyze the impact of different weight-bearing protocols on short-term complications [14,21,30]. There was no observed heterogeneity amongst the included studies (I^2^ = 0%). The pooled effect indicated no significant difference between unrestricted and restricted weight-bearing protocols (RR = 0.87, 95% CI 0.72–1.05, *p* = 0.14; 95% PI 0.58–1.31), with an absolute risk difference of −0.02%. Similarly, in the analysis of long-term complications involving three studies, no significant difference in the risk of complications was observed between the two weight-bearing protocols (RR = 1.05, 95% CI 0.70–1.57, *p* = 0.80; 95% PI 0.54–1.91), with an acceptable level of heterogeneity (I^2^ = 35%) and an absolute risk difference of 0.01% [15,28,31]. Regarding mechanical complications, only 1 study provided group-specific event counts. Specifically, mechanical failures were comparable between unrestricted weight-bearing (*n* = 389) and restricted weight-bearing (*n* = 385), including varus deformity (12 cases vs. 11 cases), screw slippage (13 cases vs. 13 cases), and non-union (4 cases vs. 5 cases). In the sensitivity analysis, after excluding the Topak study with a high risk of bias, heterogeneity was reduced to zero (I^2^ = 0%), while the pooled estimate remained non-significant (*p* = 0.84) (Supplementary Figure S2).

### 3.6. Reoperation and LOS

**Figure 4:**
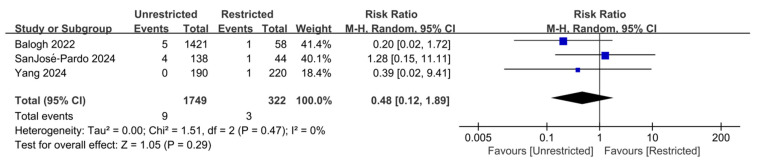
Forest plot of unadjusted relative risks for reoperation comparing unrestricted with restricted weight-bearing after hip fracture in the elderly [14,21,33]. No significant difference in reoperation risk was observed between the two weight-bearing strategies. Abbreviations: CI, confidence interval; Tau^2^, between-study variance; Chi^2^, chi-square test statistic for heterogeneity; df, degrees of freedom; I^2^, inconsistency statistic.

As shown in Figure 4, reoperation data were available from three studies [14,21,33]. Two studies further detailed the specific causes of reoperation, primarily including re-fracture (one case) and implant failure (five cases) [14,33]. Amongst these, the re-fracture and one implant failure occurred during unrestricted weight-bearing. All other events occurred during restricted weight-bearing. No evidence of heterogeneity was found between studies in the meta-analysis (I^2^ = 0%). Overall, unrestricted weight-bearing demonstrated no statistically significant difference in reoperation risk compared with restricted weight-bearing (RR = 0.48, 95% CI 0.12–1.89, *p* = 0.29; 95% PI 0.02–9.72), with an absolute risk difference of −0.01%.

**Figure 5:**
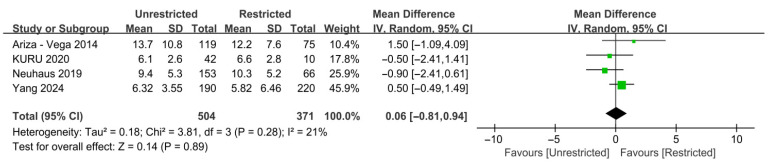
Forest plot of unadjusted mean difference for LOS comparing unrestricted with restricted weight-bearing after hip fracture in the elderly [14,29,30,34]. No significant difference in LOS was observed between the two weight-bearing strategies. Abbreviations: CI, confidence interval; LOS, length of hospital stay; Tau^2^, between-study variance; Chi^2^, chi-square test statistic for heterogeneity; df, degrees of freedom; I^2^, inconsistency statistic.

As shown in Figure 5, four studies reported LOS, with a pooled effect size of 0.06 and low heterogeneity (I^2^ = 21%) [14,29,30,34]. However, the meta-analysis found no significant difference in LOS between unrestricted weight-bearing and restricted weight-bearing (MD 0.06, 95% CI—0.81–0.94, *p* = 0.89; 95% PI—1.88–2.00).

### 3.7. Additional Analyses

Sensitivity analyses using fixed-effect models yielded consistent results (Supplementary Figures S3–S6). Due to the limited number of studies included for each outcome, subgroup analyses, meta-regression and influence diagnostics were not performed. Formal assessment of small-study effects or publication bias (such as funnel plots) was also not conducted.

### 3.8. Results of Evidence Certainty Assessment

According to the GRADE approach, there was low certainty of evidence for long-term mortality and short-term complications (Supplementary Table S7). The certainty of evidence was graded as very low for short-term mortality, long-term complications, reoperation, and length of hospital stay. No outcome was rated as moderate or high certainty.

## 4. Discussion

Considerable controversy persists regarding the optimal postoperative weight-bearing strategy for geriatric hip fracture patients. A primary concern with early full weight-bearing is the potential increase in the risk of implant failure, particularly in this osteoporotic population, which could subsequently raise reoperation and mortality rates. This systematic review and meta-analysis comprehensively evaluated the impact of different weight-bearing protocols on patient prognosis. By synthesizing data from 10 studies (including 1 RCT and 9 cohort studies), we found that an unrestricted weight-bearing protocol was associated with a significantly reduced risk of long-term all-cause mortality. However, the pooled analysis showed no significant difference in short-term mortality, postoperative complications, reoperation rates, or LOS. GRADE assessment showed low certainty of evidence for long-term mortality and short-term complications, and very low certainty of evidence for the remaining outcomes.

Regarding long-term mortality, unrestricted weight-bearing was significantly associated with a lower risk. Furthermore, the relatively narrow PI suggests that between-study heterogeneity was acceptable and that future studies are likely to demonstrate a reduction in long-term mortality with unrestricted weight-bearing. This aligns with growing evidence supporting the positive impact of early, active rehabilitation on long-term survival in this population [31,32]. Early unrestricted postoperative weight-bearing may reduce bed-rest-related complications such as pneumonia, deep vein thrombosis, urinary tract infections, and pressure injuries. Avoiding these complications may partly contribute to better long-term survival. In addition, early unrestricted postoperative weight-bearing may also promote faster recovery of limb function and independence [14,29], improving patients’ mood and quality of life, thereby indirectly enhancing overall survival. This finding resonates with the study by Pfeufer et al., which indicated that weight-bearing restrictions may impair postoperative mobility and overall recovery [13]. Nevertheless, we found that the absolute risk difference in long-term mortality between unrestricted and restricted weight-bearing was small, indicating that the magnitude of benefit may be limited. Furthermore, this association should be interpreted cautiously because postoperative weight-bearing protocols were not randomly assigned in most included studies. Patients assigned to restricted weight-bearing may have had poorer baseline health status or less stable fixation.

The preliminary meta-analysis of short-term mortality did not reveal any significant differences. Furthermore, the corresponding absolute risk difference was of limited clinical significance. However, the findings on short-term mortality were sensitive to study selection. After excluding the study by Balogh et al. [21], heterogeneity decreased substantially, and the results indicated a potential association between unrestricted weight-bearing and lower early mortality. However, this conclusion warrants cautious interpretation. Following the exclusion, the evidence base rested on only two retrospective observational studies with a limited total sample size. In addition, the extremely wide PI in the primary analysis suggests that the effect of unrestricted weight-bearing on short-term mortality may vary considerably across studies, further limiting the robustness of this finding. Therefore, this finding urgently requires validation through future well-designed, large-scale prospective studies.

Our analysis found no significant differences between the two strategies in overall complication rates, reoperation risk, or LOS. This is clinically important, as it suggests that, given stable surgical fixation, early unrestricted weight-bearing does not appear to increase the risk of early adverse events, supporting its safety. A study by Patel et al. [16] noted that whilst theoretical mechanical risks exist, clinical studies have not consistently demonstrated that restricted weight-bearing reduces such events. Our results strengthen this view, indicating that early weight-bearing is likely safe and feasible for most elderly patients treated with modern fixation. This is also consistent with existing clinical guidelines recommending early standing and weight-bearing walking training postoperatively when the patient’s condition allows [18,35]. Notably, the absolute risk difference suggested a slightly higher rate of long-term complications in the unrestricted weight-bearing group. This may partly reflect concerns regarding mechanical failures after unrestricted weight-bearing. However, the available data were insufficient to support a separate pooled analysis of mechanical complications, although this outcome is a key concern for surgeons when selecting postoperative weight-bearing protocols. In the only study with group-specific event counts, mechanical complications appeared comparable between groups, but further studies are needed to confirm this finding. Furthermore, the small number of studies, instability of the estimate, and limited absolute difference mean that this finding should not be interpreted as evidence against the safety of unrestricted weight-bearing. Furthermore, sensitivity analyses further confirmed that, after excluding the Topak study with a higher risk of bias, there was no significant difference in long-term complication risk between the two groups. Furthermore, the PIs of these second outcomes all exceed the null value, suggesting that the results of future studies may vary. However, these findings should be interpreted with caution, given the limited number of included studies.

This review concentrated on objective clinical endpoints and did not extensively analyze the effect of weight-bearing protocols on functional recovery. Existing studies in this area are limited and highly heterogeneous in terms of fracture types, assessment tools and follow-up time points, precluding meaningful quantitative synthesis. Notably, limited evidence suggests the relationship between weight-bearing and functional outcomes may be complex and potentially inconsistent with our primary findings on survival. For instance, Ariza-Vega et al. [34] reported that non-weight-bearing after surgery in patients with cervical or trochanteric fractures was independently associated with poorer Functional Independence Measure scores up to 1 year, suggesting that weight-bearing restriction may compromise long-term independence. In contrast, the RCT by Topak et al. [28] reported that partial weight-bearing led to better short-term Harris Hip Scores and mobility than full weight-bearing in patients with intertrochanteric fractures. In addition, Chen et al. [15] found no significant difference between weight-bearing as tolerated and non-weight-bearing in 12-month Harris Hip Scores among patients. These findings indicate that the functional recovery of unrestricted weight-bearing may not parallel its observed associations with long-term mortality and other safety outcomes. Therefore, a uniform weight-bearing protocol may not represent the optimal approach for this heterogeneous patient population. Future research should employ large-sample, prospective designs with standardized, multidimensional functional assessment tools (including patient-reported outcomes) to investigate the subtle effects of different weight-bearing protocols on functional recovery trajectories. Efforts should also focus on identifying which patient subgroups (e.g., based on fracture type, fixation stability, pre-injury mobility) are most likely to benefit from early unrestricted weight-bearing and which might achieve better functional outcomes with a more protective strategy. Such individualized exploration is essential for truly optimizing the overall rehabilitation pathway for geriatric hip fracture patients.

## 5. Limitations

This systematic review and meta-analysis have several limitations and the findings should be interpreted with caution. First, we rated the certainty of the evidence for all outcome measures as low or very low, which means we have limited confidence in these findings. Second, postoperative weight-bearing strategies were simplified by grouping “full weight-bearing” and “weight-bearing as tolerated” into a single “unrestricted” category. However, these strategies differ in biomechanical loading, patient autonomy, and rehabilitation intensity. Weight-bearing as tolerated is a patient-titrated protocol and may result in substantially lower actual loading than full weight-bearing, particularly in frail elderly patients. Therefore, this classification may have introduced serious misclassification bias and influenced the interpretation of the pooled estimates. However, due to the limited number of studies, further stratified or sensitivity analyses to distinguish the effects of full weight-bearing and weight-bearing as tolerated compared with restricted or partial/non-weight-bearing protocols were not feasible. Third, postoperative weight-bearing protocols were mostly not randomly assigned but were influenced by patient frailty, comorbidity burden, fracture characteristics, fixation stability, and surgeon preference. As a result, patients permitted unrestricted weight-bearing were more likely to have a more favorable baseline status and more stable fixation. Therefore, the observed association with lower mortality may reflect baseline differences rather than a true treatment effect. Fourth, clinical heterogeneity existed across studies in terms of patient characteristics, fracture types, surgical techniques, and follow-up duration, which may affect the comparability of the pooled results. However, only a limited number of studies reported adjusted estimates. Therefore, pooled results were primarily based on unadjusted effect measures, which limited the ability to control for confounding factors (such as osteoporosis, cardiovascular disease, and diabetes) and weakened causal inference. Finally, a small number of included studies for each outcome precluded meaningful subgroup analyses (fracture type, surgical technique, fixation stability), meta-regression (age, sex distribution, study design, follow-up duration), and influence diagnostics to further explore sources of heterogeneity, as well as a formal assessment of small-study effects or publication bias.

## 6. Conclusions

This meta-analysis indicates that for elderly patients with hip fractures who undergo surgical treatment, an unrestricted weight-bearing strategy may be associated with a lower risk of long-term all-cause mortality, without increasing short-term mortality, complication rate, reoperation rate, or LOS. These findings indicate that unrestricted weight-bearing may be a feasible postoperative rehabilitation approach in selected patients. However, these findings should be interpreted with caution. Further well-designed prospective studies are required to confirm these findings.

## Figures and Tables

**Table 1:** Characteristics of included studies.

Study	Publication Date, y	Country	Study Design	No. of Patients	Age, Mean (SD), y	Sex, %, Female	Fracture Type (*n*)	Surgical Procedure (*n*)	Protocol of Weight-Bearing
Topak et al. [28]	2023	Turkey	RCT	85	76.67 (8.62)	49.40%	Intertrochanteric femur fractures (85)	Proximal intramedullary nailing (85)	Full weight-bearing; Partial weight-bearing
KURU et al. [29]	2020	Turkey	Retrospective cohort study	52	82.9 (6.5)	69.20%	Intertrochanteric fractures (38); Femoral neck fractures (14)	Partial prosthesis (52)	Full weight-bearing; Partial weight-bearing
Balogh et al. [21]	2022	Australia	Retrospective cohort study	1479	Full weight-bearing: 83.8 (7.9); Restricted/non-weight-bearing: 81.3 (9.4)	Full weight-bearing: 70%; Restricted/non-weight-bearing: 59%	Intracapsular undisplaced/impacted (367); Intracapsular displaced (311); Peritrochanteric (incl. basicervical) (736); Subtrochanteric (65)	Cannulated screws (129); Cemented hemiarthroplasty (427); Uncemented hemiarthroplasty (28); Long femoral IM nail (246); Short femoral IM nail (504); Sliding hip screw (55); Cemented total hip replacement (78); Uncemented total hip replacement (3); Other (9)	Weight-bearing as tolerated; Restricted/non-weight-bearing
Yang et al. [14]	2024	China	Retrospective cohort study	410	Immediate weight-bearing as tolerated: 80.97 (7.56); Delayed weight-bearing: 80.91 (7.28)	Immediate weight-bearing as tolerated: 68.9%; Delayed weight-bearing: 73.6%	Intertrochanteric fractures (410)	Intramedullary fixation (410)	Weight-bearing as tolerated; Non-weight-bearing
Chen et al. [15]	2021	China	Retrospective cohort study	806	77.8 (7.6)	74.80%	Intertrochanteric fractures (806)	Intramedullary fixation (806)	Weight-bearing as tolerated; Non-weight-bearing
Neuhaus et al. [30]	2019	Switzerland	Retrospective cohort study	219	83 (7.1)	68%	Femoral neck (62); Trochanteric fractures (157)	Intramedullary nail (158); Total hip replacement (35); Hemiarthroplasty (26)	Full weight-bearing; Partial weight-bearing
Dubin et al. [31]	2021	Israel	Retrospective cohort study	2104	Full weight-bearing : 78.82 (12.5); Partial weight-bearing : 79.48 (9.6); Non-weight-bearing: 83.07 (8.14)	Full weight-bearing: 75.3%; Partial weight-bearing: 70.1%; Non-weight-bearing: 66.8%	Subcapital fractures (787)^#^; Pertrochanteric fractures (882) ^#^; Basicervical fractures (146) ^#^; Midcervical fractures (22) ^#^; Subtrochanteric fractures (87) ^#^;	Conservative (7) ^#^; Cannulated screws (234) ^#^; Proximal femoral nail (413) ^#^; Thompson hemiarthroplasty (475) ^#^; Richard’s nail (534) ^#^; Dynamic hip screw (117) ^#^; Trochanteric antegrade nail (128) ^#^; Bipolar hemiarthroplasty (68) ^#^; Percutaneous compression plate (16) ^#^	Full weight-bearing; Partial weight-bearing Non-weight-bearing
Jiménez-Moleón et al. [32]	2015	Spain	Prospective cohort study	275	81.4 (6.8)	79%	Intracapsular fractures (cervical) (129) *; Extracapsular (inter- or subtrochanteric) fractures (143) *	Dynamic hip screw with plate (133) *; Intramedullary hip screw (46) *; Hemiarthroplasty (93) *	Weight-bearing as tolerated; Non-weight-bearing
SanJosé-Pardo et al. [33]	2024	Spain	Prospective cohort study	182	85.53	84%	Subtrochanteric fractures (182)	Cephalomedullary nailing (182)	Weight-bearing as tolerated; Restricted/non-weight-bearing
Ariza-Vega et al. [34]	2014	Spain	Prospective cohort study	194	81.4 (6.1)	81%	Cervical (femoral neck) fractures (91); Trochanteric fractures (103)	Dynamic hip screw with plate (96); Intramedullary hip screw (33); Hemiarthroplasty (65)	Full weight-bearing; Non-weight-bearing

^#^ Total number < 2104 because of missing data. * Total number < 275 because of missing data.

**Table 2:** Quality analysis and risk of bias assessment.

Newcastle-Ottawa	Selection				Comparability	Outcome			Total Score (9/9)
Study	Representativeness of Exposed Cohort	Selection of Nonexposed Cohort	Ascertainment of Exposure	Outcome of Interest Not Present at Start of Study		Assessment of Outcome	Sufficient Follow-Up Time	Adequacy of Follow-Up	
KURU et al. [29]	*	*	*	*	*	*	-	*	7/9
Balogh et al. [21]	*	*	*	*	**	*	*	*	9/9
Yang et al. [14]	*	*	*	*	**	*	*	*	9/9
Chen et al. [15]	*	*	*	*	**	*	*	*	9/9
Neuhaus et al. [30]	*	*	*	*	*	*	-	-	6/9
Dubin et al. [31]	*	*	*	*	*	*	*	-	7/9
Jiménez-Moleón et al. [32]	*	*	*	*	**	*	*	*	9/9
SanJosé-Pardo et al. [33]	*	*	*	*	*	*	*	*	8/9
Ariza-Vega et al. [34]	*	*	*	*	*	*	*	-	7/9
**Cochrane risk of** **bias tool**									
**Study**	**Randomization process**	**Deviations from intended interventions**		**Missing outcome data**	**Measurement of the outcome**		**Selection of the reported result**		**Overall** **Risk**
Topak et al. [28]	Some concerns	Some concerns		High risk	Some concerns		Low risk		High risk

- indicates no point awarded. * indicates one point awarded. ** indicates two points awarded.

## Data Availability

All data supporting the findings of this study are included within the published article and its Supplementary Materials. Additional information can be obtained from the corresponding authors upon reasonable request.

## Supplementary Materials

The following supporting information can be downloaded at: https://www.mdpi.com/article/10.3390/jcm15103912/s1, Table S1: PRISMA checklist; Table S2: MEDLINE search criteria; Table S3: Embase search criteria; Table S4: Cochrane Library search criteria; Table S5: Web of Science search criteria; Table S6: Inclusion of complication types; Table S7: Certainty of findings according to GRADE; Figure S1: Leave-one-out sensitivity analysis for short-term mortality; Figure S2: Leave-one-out sensitivity analysis for long-term complications; Figure S3: Fixed-effect model sensitivity analysis for mortality; Figure S4: Fixed-effect model sensitivity analysis for complications; Figure S5: Fixed-effect model sensitivity analysis for reoperation; Figure S6: Fixed-effect model sensitivity analysis for LOS.
