# Supplementary material for: Impact of Postoperative Weight-Bearing Protocols on Prognosis in Geriatric Hip Fracture Patients: A Systematic Review and Meta-Analysis
Source: J Clin Med. 2026 May 19;15(10):3912. doi: 10.3390/jcm15103912 (PMC13207775; doi:10.3390/jcm15103912)
Supplement: Supplementary file 1 [file jcm-15-03912-s001.zip › Sumplement.pdf]

Title: Impact of Postoperative Weight-Bearing Protocols on prognosis in Geriatric Hip Fracture Patients: A Systematic Review and Meta-Analysis

Table S1. PRISMA checklist
Table S2. MEDLINE search criteria
Table S3. Embase search criteria
Table S4. Cochrane Library search criteria
Table S5. Web of Science search criteria.
Table S6. Inclusion of complication types.
Table S7. Certainty of findings according to GRADE.
Figure S1. Leave-one-out sensitivity analysis for short-term mortality.
Figure S2. Leave-one-out sensitivity analysis for long-term complications.
Figure S3. Fixed-effect model sensitivity analysis for mortality.
Figure S4. Fixed-effect model sensitivity analysis for complications.
Figure S5. Fixed-effect model sensitivity analysis for reoperation.
Figure S6. Fixed-effect model sensitivity analysis for LOS.

Table S1. PRISMA Checklist

Section and Topic	Item #	Checklist item	Reported on page
**TITLE**			
Title	1	Identify the report as a systematic review.	Title Page
**ABSTRACT**			
Abstract	2	See the PRISMA 2020 for Abstracts checklist.	Methods Page
**INTRODUCTION**			
Rationale	3	Describe the rationale for the review in the context of existing knowledge.	Introduction Paragraph
Objectives	4	Provide an explicit statement of the objective(s) or question(s) the review addresses.	Introduction Paragraph
**METHODS**			
Eligibility criteria	5	Specify the inclusion and exclusion criteria for the review and how studies were grouped for the syntheses.	Methods Paragraph
Information sources	6	Specify all databases, registers, websites, organisations, reference lists and other sources searched or consulted to identify studies. Specify the date when each source was last searched or consulted.	Methods Paragraph
Search strategy	7	Present the full search strategies for all databases, registers and websites, including any filters and limits used.	Supplementary Appendix
Selection process	8	Specify the methods used to decide whether a study met the inclusion criteria of the review, including how many reviewers screened each record and each report retrieved, whether they worked independently, and if applicable, details of automation tools used in the process.	Methods Paragraph
Data collection process	9	Specify the methods used to collect data from reports, including how many reviewers collected data from each report, whether they worked independently, any processes for obtaining or confirming data from study investigators, and if applicable, details of automation tools used in the process.	Methods Paragraph

Section and Topic	Item #	Checklist item	Reported on page
Data items	10a	List and define all outcomes for which data were sought. Specify whether all results that were compatible with each outcome domain in each study were sought (e.g. for all measures, time points, analyses), and if not, the methods used to decide which results to collect.	Methods Paragraph
	10b	List and define all other variables for which data were sought (e.g. participant and intervention characteristics, funding sources). Describe any assumptions made about any missing or unclear information.	Methods Paragraph
Study risk of bias assessment	11	Specify the methods used to assess risk of bias in the included studies, including details of the tool(s) used, how many reviewers assessed each study and whether they worked independently, and if applicable, details of automation tools used in the process.	Methods Paragraph
Effect measures	12	Specify for each outcome the effect measure(s) (e.g. risk ratio, mean difference) used in the synthesis or presentation of results.	Methods Paragraph
Synthesis methods	13a	Describe the processes used to decide which studies were eligible for each synthesis (e.g. tabulating the study intervention characteristics and comparing against the planned groups for each synthesis (item #5)).	Methods Paragraph
	13b	Describe any methods required to prepare the data for presentation or synthesis, such as handling of missing summary statistics, or data conversions.	Methods Paragraph
	13c	Describe any methods used to tabulate or visually display results of individual studies and syntheses.	Methods Paragraph
	13d	Describe any methods used to synthesize results and provide a rationale for the choice(s). If meta-analysis was performed, describe the model(s), method(s) to identify the presence and extent of statistical heterogeneity, and software package(s) used.	Methods Paragraph
	13e	Describe any methods used to explore possible causes of heterogeneity among study results (e.g. subgroup analysis, meta-regression).	Methods Paragraph
	13f	Describe any sensitivity analyses conducted to assess robustness of the synthesized results.	Methods Paragraph
Reporting bias assessment	14	Describe any methods used to assess risk of bias due to missing results in a synthesis (arising from reporting biases).	Methods Paragraph
Certainty assessment	15	Describe any methods used to assess certainty (or confidence) in the body of evidence for an outcome.	Methods Paragraph

Section and Topic	Item #	Checklist item	Reported on page
**RESULTS**			
Study selection	16a	Describe the results of the search and selection process, from the number of records identified in the search to the number of studies included in the review, ideally using a flow diagram.	Results Paragraph
	16b	Cite studies that might appear to meet the inclusion criteria, but which were excluded, and explain why they were excluded.	Results Paragraph
Study characteristics	17	Cite each included study and present its characteristics.	Results Paragraph
Risk of bias in studies	18	Present assessments of risk of bias for each included study.	Supplementary Appendix
Results of individual studies	19	For all outcomes, present, for each study: (a) summary statistics for each group (where appropriate) and (b) an effect estimate and its precision (e.g. confidence/credible interval), ideally using structured tables or plots.	Results Paragraph
Results of syntheses	20a	For each synthesis, briefly summarise the characteristics and risk of bias among contributing studies.	Results Paragraph
	20b	Present results of all statistical syntheses conducted. If meta-analysis was done, present for each the summary estimate and its precision (e.g. confidence/credible interval) and measures of statistical heterogeneity. If comparing groups, describe the direction of the effect.	Results Paragraph
	20c	Present results of all investigations of possible causes of heterogeneity among study results.	Results Paragraph
	20d	Present results of all sensitivity analyses conducted to assess the robustness of the synthesized results.	-
Reporting biases	21	Present assessments of risk of bias due to missing results (arising from reporting biases) for each synthesis assessed.	Supplementary Appendix
Certainty of evidence	22	Present assessments of certainty (or confidence) in the body of evidence for each outcome assessed.	Supplementary Appendix
**DISCUSSION**			
Discussion	23a	Provide a general interpretation of the results in the context of other evidence.	Discussion Paragraph

Section and Topic	Item #	Checklist item	Reported on page
	23b	Discuss any limitations of the evidence included in the review.	Discussion Paragraph
	23c	Discuss any limitations of the review processes used.	Discussion Paragraph
	23d	Discuss implications of the results for practice, policy, and future research.	Discussion Paragraph
**OTHER INFORMATION**			
Registration and protocol	24a	Provide registration information for the review, including register name and registration number, or state that the review was not registered.	Methods Paragraph
	24b	Indicate where the review protocol can be accessed, or state that a protocol was not prepared.	Methods Paragraph
	24c	Describe and explain any amendments to information provided at registration or in the protocol.	-
Support	25	Describe sources of financial or non-financial support for the review, and the role of the funders or sponsors in the review.	-
Competing interests	26	Declare any competing interests of review authors.	-
Availability of data, code and other materials	27	Report which of the following are publicly available and where they can be found: template data collection forms; data extracted from included studies; data used for all analyses; analytic code; any other materials used in the review.	-

From: Page MJ, McKenzie JE, Bossuyt PM, Boutron I, Hoffmann TC, Mulrow CD, et al. The PRISMA 2020 statement: an updated guideline for reporting systematic reviews. BMJ 2021;372:n71. doi: 10.1136/bmj.n71. This work is licensed under CC BY 4.0. To view a copy of this license, visit https://creativecommons.org/licenses/by/4.0/

Table S2. MEDLINE search criteria

#	Searches
1	"Hip Fractures"[Mesh]
2	((((((((((((("Intertrochanteric Fracture*") OR ("Intertrochanteric Femur Fracture*")) OR ("Subtrochanteric Fracture*")) OR ("Subtrochanteric Femoral Fracture*")) OR ("Subtrochanteric Femur Fracture*")) OR ("Trochanteric Fracture*")) OR ("Femoral Trochanter Fracture*")) OR ("Femur Trochanter Fracture*")) OR ("Trochanteric Femur Fracture*")) OR ("Femoral Neck Fracture*")) OR ("Femur Neck Fracture*")) OR ("Hip Fracture*")) OR ("Broken Hip")) OR ("Fractured Hip")
3	(((((((((((((("Intertrochanteric Fracture*") OR ("Intertrochanteric Femur Fracture*")) OR ("Subtrochanteric Fracture*")) OR ("Subtrochanteric Femoral Fracture*")) OR ("Subtrochanteric Femur Fracture*")) OR ("Trochanteric Fracture*")) OR ("Femoral Trochanter Fracture*")) OR ("Femur Trochanter Fracture*")) OR ("Trochanteric Femur Fracture*")) OR ("Femoral Neck Fracture*")) OR ("Femur Neck Fracture*")) OR ("Hip Fracture*")) OR ("Broken Hip")) OR ("Fractured Hip")) OR ("Hip Fractures"[Mesh])
4	"Weight-Bearing"[Mesh]
5	((((((((((("Weight Bearing*") OR ("Weightbearing*")) OR ("Weight-Bearing*")) OR ("Load Bearing*")) OR ("Loadbearing*")) OR ("Load-Bearing*")) OR ("Load carrying*")) OR ("Axial Loading*")) OR ("AxialLoading*")) OR ("Axial-Loading*")) OR ("Loadcarrying*")) OR ("Load-carrying*")
6	(((((((((((("Weight Bearing*") OR ("Weightbearing*")) OR ("Weight-Bearing*")) OR ("Load Bearing*")) OR ("Loadbearing*")) OR ("Load-Bearing*")) OR ("Load carrying*")) OR ("Axial Loading*")) OR ("AxialLoading*")) OR ("Axial-Loading*")) OR ("Loadcarrying*")) OR ("Load-carrying*")) OR ("Weight-Bearing"[Mesh])
7	((((((((((((("Weight Bearing*") OR ("Weightbearing*")) OR ("Weight-Bearing*")) OR ("Load Bearing*")) OR ("Loadbearing*")) OR ("Load-Bearing*")) OR ("Load carrying*")) OR ("Axial Loading*")) OR ("AxialLoading*")) OR ("Axial-Loading*")) OR ("Loadcarrying*")) OR ("Load-carrying*")) OR ("Weight-Bearing"[Mesh])) AND ((((((((((((((("Intertrochanteric Fracture*") OR ("Intertrochanteric Femur Fracture*")) OR ("Subtrochanteric Fracture*")) OR ("Subtrochanteric Femoral Fracture*")) OR ("Subtrochanteric Femur Fracture*")) OR ("Trochanteric Fracture*")) OR ("Femoral Trochanter Fracture*")) OR ("Femur Trochanter Fracture*")) OR ("Trochanteric Femur Fracture*")) OR ("Femoral Neck Fracture*")) OR ("Femur Neck Fracture*")) OR ("Hip Fracture*")) OR ("Broken Hip")) OR ("Fractured Hip")) OR ("Hip Fractures"[Mesh]))

Table S3. Embase search criteria

#	Searches
1	'hip fracture'/exp
2	'intertrochanteric fracture*' OR 'intertrochanteric femur fracture*' OR 'subtrochanteric fracture*' OR 'subtrochanteric femoral fracture*' OR 'subtrochanteric femur fracture*' OR 'trochanteric fracture*' OR 'femoral trochanter fracture*' OR 'femur trochanter fracture*' OR 'trochanteric femur fracture*' OR 'femoral neck fracture*' OR 'femur neck fracture*' OR 'hip fracture*' OR 'broken hip' OR 'fractured hip'
3	#1 OR #2
4	'weight bearing'/exp
5	weight bearing*' OR 'load-bearing*' OR 'load bearing*' OR 'loadbearing*' OR 'load carrying*' OR 'weightbearing*' OR 'axial loading*' OR 'axialloading*' OR 'weight-bearing*' OR 'loadcarrying*' OR 'load-carrying*' OR 'axial-loading*'
6	#4 OR #5
7	#3 AND #6

Table S4. Cochrane Library search criteria

#	Searches
1	MeSH descriptor: [Hip Fractures] explode all trees
2	(Intertrochanteric Fracture*) OR (Intertrochanteric Femur Fracture*) OR (Subtrochanteric Fracture*) OR (Subtrochanteric Femoral Fracture*) OR (Subtrochanteric Femur Fracture*) OR (Trochanteric Fracture*) OR (Femoral Trochanter Fracture*) OR (Femur Trochanter Fracture*) OR (Trochanteric Femur Fracture*) OR (Femoral Neck Fracture*) OR (Femur Neck Fracture*) OR (Hip Fracture*) OR (Broken Hip) OR (Fractured Hip)
3	#1 OR #2
4	MeSH descriptor: [Weight-Bearing] explode all trees
5	((Weight Bearing) OR (Load-Bearing) OR (Load Bearing) OR (Loadbearing) OR (Load carrying) OR (Weightbearing) OR (Axial Loading) OR (Axial Loadings) OR (Weight-Bearing))
6	#4 OR #5
7	#3 AND #6

Table S5. Web of Science search criteria.

#	Searches
1	Intertrochanteric Fracture* OR Intertrochanteric Femur Fracture* OR Subtrochanteric Fracture* OR Subtrochanteric Femoral Fracture* OR Subtrochanteric Femur Fracture* OR Trochanteric Fracture* OR Femoral Trochanter Fracture* OR Femur Trochanter Fracture* OR Trochanteric Femur Fracture* OR Femoral Neck Fracture* OR Femur Neck Fracture* OR Hip Fracture* OR Broken Hip OR Fractured Hip (All Fields)
2	Weight Bearing* OR Load-Bearing* OR Load Bearing* OR Loadbearing* OR Load carrying* OR Weightbearing* OR Axial Loading* OR AxialLoading* OR Weight-Bearing* OR Loadcarrying* OR Load-carrying* OR Axial-loading* (All Fields)
3	#1 AND #2

Table S6. Inclusion of complication types.

Study	Short-term complications	Long-term complications
Balogh et al.	Pulmonary embolus, deep vein thrombosis, lower respiratory tract infection, delirium, urinary tract infection, and a post-operative fall on the ward as an inpatient.	-
Neuhaus et al.	Deep vein throm bosis, pulmonary embolism, urinary tract infection, decubitus, gastroduodenal ulcers, pseudomembranous colitis, urosepsis, electrolyte dysregulation requiring treatment, delirium, renal insuffificiency, pneumonia including aspiration pneumonia, heart and respiratory insuffificiency, myocardial infarction, ileus, wound infection, transient ischemic attack or stroke, and death.	-
Yang et al.	Deep vein thrombosis, pulmonary embolism, pneumonia, urinary tract infection, delirium, neurological complications, cardiovascular complications	-
Chen et al.	-	Hip varus deformity, screw cutout, stress fracture of femoral shaft, and nonunion
Dubin et al.	-	Cerebrovascular accident, pulmonary embolism, deep vein thrombosis.
Topak et al.	-	Screw cut-out, Z-effect, implant failure, infection.

Table S7. Certainty of findings according to GRADE.

Outcome	Studies	No. of patients	Effect estimate (95% CI)	Certainty of the evidence (GRADE)
Short-term mortality	3 observational studies	1,750	RR = 0.58, 95% CI 0.14 - 2.34	⊕◯◯◯ Very low[a,b,c,d,e]
Long-term mortality	3 observational studies	2,789	RR = 0.67, 95% CI 0.51 - 0.88	⊕⊕◯◯ Low[a,b,f]
Short-term complications	3 observational studies	2,108	RR = 0.87, 95% CI 0.72 - 1.05	⊕⊕◯◯ Low[a,b,d]
Long-term complications	2 observational studies and 1 RCT	2,963	RR = 1.05, 95% CI 0.70 - 1.57	⊕◯◯◯ Very low[a,b,d,g]
Reoperation	3 observational studies	2,071	RR = 0.48, 95% CI 0.12 - 1.89	⊕◯◯◯ Very low[a,b,d,e]
LOS	4 observational studies	875	MD = 0.06, 95% CI -0.81 - 0.94	⊕◯◯◯ Very low[a,b,d,h]

Abbreviation: RR, risk ratio; MD, mean difference; CI, confidence interval; LOS, length of hospital stay.

GRADE Working Group grades of evidence

High certainty: we are very confident that the true effect lies close to that of the estimate of the effect.

Moderate certainty: we are moderately confident in the effect estimate: the true effect is likely to be close to the estimate of the effect, but there is a possibility that it is substantially different.

Low certainty: our confidence in the effect estimate is limited: the true effect may be substantially different from the estimate of the effect.

Very low certainty: we have very little confidence in the effect estimate: the true effect is likely to be substantially different from the estimate of effect.

Explanations:

a. Most included studies were observational studies; therefore, the initial certainty of evidence was rated as low.

b. Fewer than 10 studies were included in the analysis, and publication bias could not be formally assessed.

c. There was substantial statistical heterogeneity.

d. The confidence interval was wide or crossed the null value, indicating imprecision.

e. The prediction interval was wide.

f. Although the association was statistically significant, the pooled estimate was mainly based on unadjusted data and residual confounding could not be excluded.

g. One included RCT had a high risk of bias.

h. LOS may be influenced by differences in healthcare systems, discharge pathways, and rehabilitation availability.

Figure S1. Leave-one-out sensitivity analysis for short-term mortality.

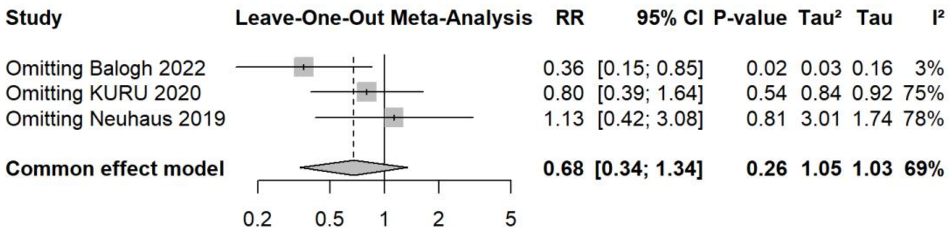

Abbreviation: RR, risk ratio; CI, confidence interval; Tau^2, between-study variance; Tau, square root of between-study variance; I^2, inconsistency statistic.

Figure S2. Leave-one-out sensitivity analysis for long-term complications.

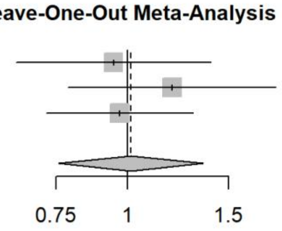

Study	RR	95% CI	P-value	Tau^2	Tau	I^2
Omitting Chen 2021	0.95	[0.64; 1.40]	0.78	0.59	0.77	64%
Omitting Dubin 2021	1.20	[0.79; 1.82]	0.40	0.29	0.54	47%
Omitting Topak 2023	0.97	[0.72; 1.30]	0.84	0	0	0%
**Common effect model**	**1.01**	**[0.76; 1.35]**	**0.93**	**< 0.01**	**< 0.01**	**35%**

Abbreviation: RR, risk ratio; CI, confidence interval; Tau^2, between-study variance; Tau, square root of between-study variance; I^2, inconsistency statistic.

Figure S3. Fixed-effect model sensitivity analysis for mortality.

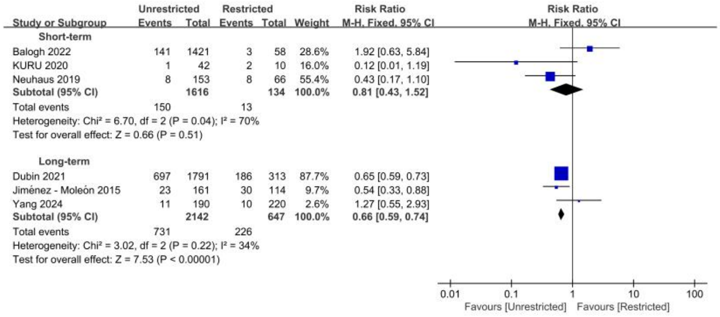

Abbreviation: CI, confidence interval; Chi^2, chi-square test statistic for heterogeneity; df, degrees of freedom; I^2, inconsistency statistic.

Figure S4. Fixed-effect model sensitivity analysis for complications.

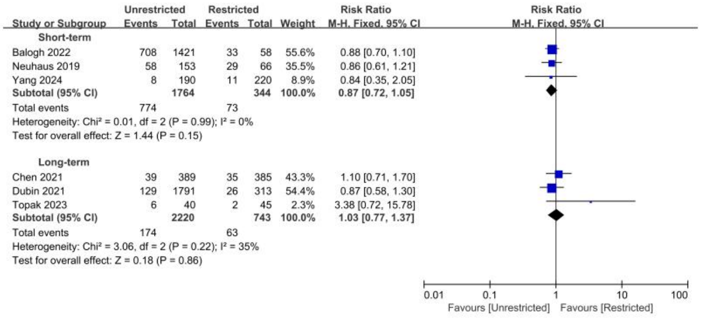

Abbreviation: CI, confidence interval; Chi^2, chi-square test statistic for heterogeneity; df, degrees of freedom; I^2, inconsistency statistic.

Figure S5. Fixed-effect model sensitivity analysis for reoperation.

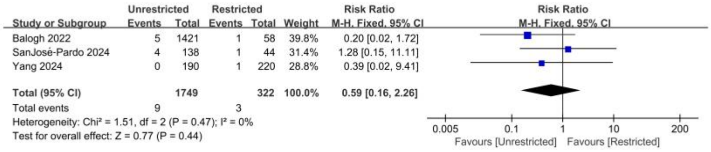

Abbreviation: CI, confidence interval; Chi^2, chi-square test statistic for heterogeneity; df, degrees of freedom; I^2, inconsistency statistic.

Figure S6. Fixed-effect model sensitivity analysis for LOS.

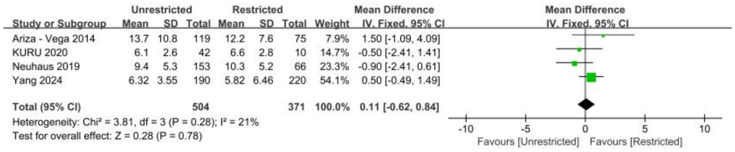

Abbreviation: CI, confidence interval; LOS, length of hospital stay; Chi^2, chi-square test statistic for heterogeneity; df, degrees of freedom; I^2, inconsistency statistic.
