# Supplementary figures and images for: Impact of Postoperative Weight-Bearing Protocols on Prognosis in Geriatric Hip Fracture Patients: A Systematic Review and Meta-Analysis
Source: J Clin Med. 2026 May 19;15(10):3912. doi: 10.3390/jcm15103912 (PMC13207775; doi:10.3390/jcm15103912)

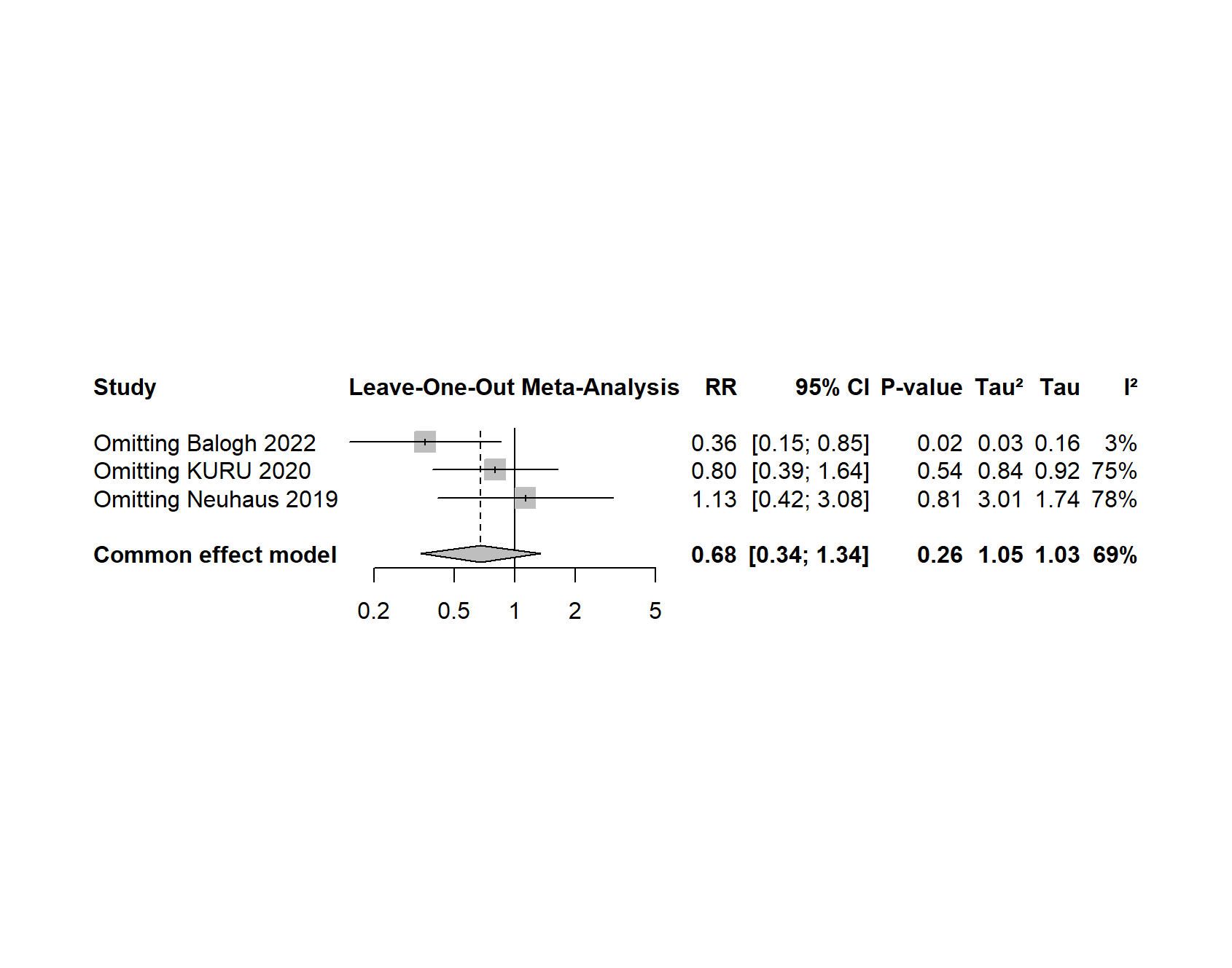

Supplement: Supplementary file 1 [file jcm-15-03912-s001.zip › Figure S1.tiff]

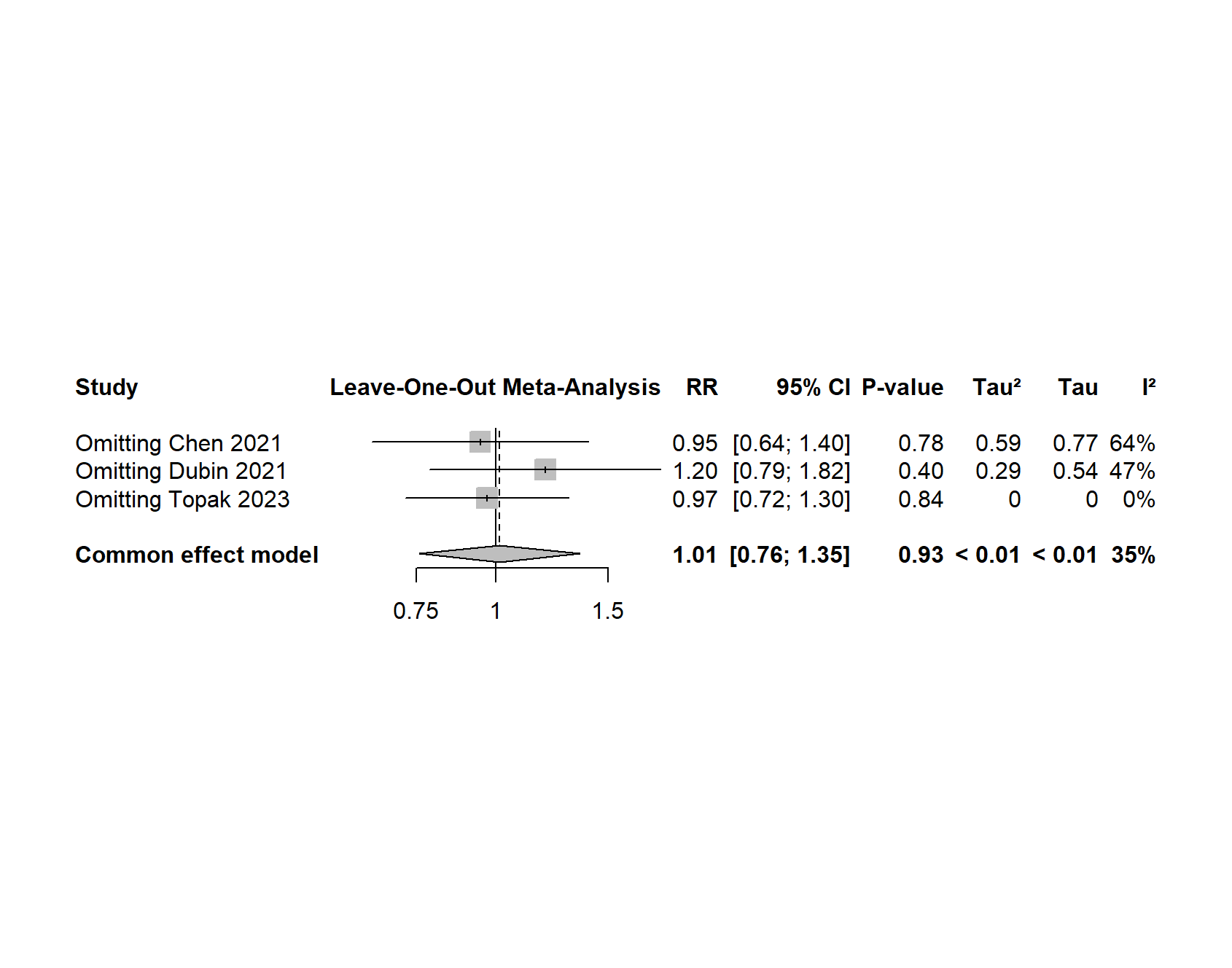

Supplement: Supplementary file 1 [file jcm-15-03912-s001.zip › Figure S2.tiff]
